# Basic life support: evaluation of learning using simulation and immediate feedback devices^1^

**DOI:** 10.1590/1518-8345.1957.2942

**Published:** 2017-10-30

**Authors:** Lucia Tobase, Heloisa Helena Ciqueto Peres, Edenir Aparecida Sartorelli Tomazini, Simone Valentim Teodoro, Meire Bruna Ramos, Thatiane Facholi Polastri

**Affiliations:** 2 PhD, RN, Serviço de Atendimento Móvel de Urgências (SAMU), São Paulo, SP, Brazil.; 3 PhD, Full Professor, Escola de Enfermagem, Universidade de São Paulo, São Paulo, SP, Brazil.; 4 Master’s student, Escola de Enfermagem, Universidade de São Paulo, São Paulo, SP, Brazil. RN, Serviço de Atendimento Móvel de Urgências (SAMU), São Paulo, SP, Brazil.; 5 Emergency Specialist, RN, Serviço de Atendimento Móvel de Urgências (SAMU), São Paulo, SP, Brazil.; 6 Specialist in Cardiology Nursing, RN, Instituto do Coração (InCor), Hospital das Clínicas, Faculdade de Medicina, Universidade de São Paulo, São Paulo, SP, Brazil.

**Keywords:** Education Nursing, Education Distance, Educational Technology, Cardiopulmonary Resuscitation, Simulation, Basic Cardiac Life Support

## Abstract

**Objective:**

to evaluate students’ learning in an online course on basic life support with immediate feedback devices, during a simulation of care during cardiorespiratory arrest.

**Method:**

a quasi-experimental study, using a before-and-after design. An online course on basic life support was developed and administered to participants, as an educational intervention. Theoretical learning was evaluated by means of a pre- and post-test and, to verify the practice, simulation with immediate feedback devices was used.

**Results:**

there were 62 participants, 87% female, 90% in the first and second year of college, with a mean age of 21.47 (standard deviation 2.39). With a 95% confidence level, the mean scores in the pre-test were 6.4 (standard deviation 1.61), and 9.3 in the post-test (standard deviation 0.82, p <0.001); in practice, 9.1 (standard deviation 0.95) with performance equivalent to basic cardiopulmonary resuscitation, according to the feedback device; 43.7 (standard deviation 26.86) mean duration of the compression cycle by second of 20.5 (standard deviation 9.47); number of compressions 167.2 (standard deviation 57.06); depth of compressions of 48.1 millimeter (standard deviation 10.49); volume of ventilation 742.7 (standard deviation 301.12); flow fraction percentage of 40.3 (standard deviation 10.03).

**Conclusion:**

the online course contributed to learning of basic life support. In view of the need for technological innovations in teaching and systematization of cardiopulmonary resuscitation, simulation and feedback devices are resources that favor learning and performance awareness in performing the maneuvers.

## Introduction

In the world scene, cardiopulmonary arrest (CPA^1^, defined as the cessation of cardiac mechanical activity and confirmed by the absence of circulation signals^2^, is considered a public health problem, especially when it occurs in an out-of-hospital environment. The most important determinant for survival is the presence of an individual to perform cardiopulmonary resuscitation (CPR) maneuvers: a health professional, who can apply resuscitation skills adapted to different circumstances, or lay people trained in basic life support (BLS), in the case of CPR occurring outside the health service environment^3^.

The BLS is considered the basis for care in cases of CPR, and defines the primary sequence of resuscitation that save lives, including immediate recognition of the condition, activation of the emergency response system, early CPR and rapid defibrillation^4^. In the advanced life support (ALS), interventions are based on basic support previously initiated in order to increase the probability of return to spontaneous circulation, with medication therapy, advanced management of airways, and physiological monitoring with equipment and devices. After spontaneous circulation returns, neurological survival and evolution can be improved with post-CPR care^5^.

However, although some advanced support techniques improve survival, the basic support interventions are determinant in increasing survival rates; the success of resuscitation depends primarily on the effectiveness of the initial actions. These questions refer to the reflection on the undervaluation of BLS learning, from the perception of health professionals, who consider learning about ALS more important than that of basic support.

In a more comprehensive manner, one of the greatest challenges is to increase the access to teaching of CPR maneuvers, including in Brazil, thus minimizing the time between life support and defibrillation, and to establish processes for the continuous improvement of resuscitation quality^1^.

In this context, in an active, affective and collaborative way, the simulation has occupied a prominent place, from professional formation to education during the professional life, with association of technological resources, based on active methodologies. As a representation of the real-life event applied in a controlled environment, the use of simulation aims to provide safe care, enabling students and health professionals to practice and reflect on the skills^6^. 

Teaching with simulation, and *debriefing,* is not limited to specific clinical laboratory encounters. It is possible to use case studies in the classroom, with discussion supported in virtual simulations; to propose *in situ* simulations^7^ with actors, standardized patients or simulators, with or without recording of the activity for *debriefing* during discussion of the didactic content; assess the learning needs and fill performance gaps in different scenarios; to provide the monitoring and (re)planning of activities, enabling the educator and/or employer to evaluate the competencies of the student and/or recently hired professional in the organization^8^.

For the coherent orientation of the strategy, organizations such as the *International Nursing Association for Clinical Simulation and Learning* (INACSL) provide guidelines on terminology, integrity of participants, outline of objectives, role of facilitator, *debriefing*, evaluation of participants and activity^9^.

According to the guidelines of the American Heart Association (AHA/2015), in addition to the simulation, the use of technology in the management of CPR is emphasized, aiming at rapid action, valuation of adequate training, and coordinated efforts to increase the chances of post-arrest survival^10^. 

In professional education and training, short-term online courses are recommended for teaching and maintaining proper resuscitation maneuvers. Technological resources, such as immediate feedback electronic devices, can be used to monitor CPR^6^, online or during real-time training.

The feedback devices are resources that allow the monitoring of performance in performing CPR, in relation to several parameters, such as compression ratio and depth, flow fraction, frequency and volume of ventilation, among others. According to the equipment used, different parameters are provided to be used as indicators of quality in the analysis of CPR administration. The devices range from the most common, such as metronomes, to the more complex ones, such as defibrillators and simulators, equipped with software and pressure detection sensors for evaluation of compressions and ventilations^11^.

Considering that BLS skills seem to be learned with the same ease by self-learning (video or computer) and by practice, compared to traditional classes provided by instructors^11^, and seeking to combine educational technological resources on resuscitation maneuvers, the objective of this study was to evaluate the learning of the students participating in an online BLS course with the use of immediate feedback devices. The hypothesis of the study was that the online course contributes to incremental BLS learning.

## Method

This was a quasi-experimental study, of a before--and-after design, about the application of an online course as an educational intervention, to evaluate the BLS learning of undergraduate nursing students at a public university in the city of São Paulo, between 2014 and 2015. The research was approved by the Ethics Committee in Research of the university in question, according to protocol nº 526.932, identifier CAAE 27029214.4.0000.5392.

For selection of participants, an invitation and the Terms of Free and Informed Consent were sent, by electronic mail, to all 283 of the first- to fourth-year nursing students. Interested respondents received the link and password to access the online course.

The inclusion criterion was: being regularly enrolled in the baccalaureate course in Nursing, in 2014. As an exclusion criterion, non-compliance with any of the phases of the online course, such as: pre-testing, virtual classroom attendance, post-test, participation in the practical activity simulation, and evaluation of the online course. In a convenience sample, 94 students agreed to participate, 88 accessed the virtual environment, 67 finished the theoretical part, and 62 completed the online course.

The following instruments were used for data collection: a) pre-/post-test, composed of 20 objective questions, with scores of 0.5 or 0, structured according to the tests applied in the BLS, and which were previously analyzed by eight experts; B) 20-item checklist, with a score equivalent to 0.5 or 0, based on the BLS evaluation tool and the professional experience of the researcher, previously analyzed by five specialists; C) immediate-feedback mobile electronic devices (*Resusci Anne QCPR* ^®^ with *SkillReporter* ^®^, *SimPad* ^®^ e *SkillGuide* ^®^ *software*) to monitor the compressions of resuscitation (rhythm, depth, frequency, chest release at each compression) and ventilations (frequency, volume). In the result of the resuscitation performance, the percentage of performance score is indicated: from 0 to 49% (basic performance of CPR); from 50 to 74% (intermediate performance of CPR); from 75 to 100% (advanced performance of CPR)^12^.

In the development of the online course on BLS, with a workload of 20 hours, we used the analysis, design, development, implementation, evaluation (ADDIE) model of instructional design, based on Andragogy^13^ and the Significant Learning Theory^14^. The educational objectives were structured based on the Bloom taxonomy, regarding the actions required to identify CPR in the adult and perform the BLS maneuvers, orienting the design of the instructional matrix and storyboards to organize the sequence of content and development of the learning objects, such as virtual classes, interactive exercises, videos, and an infographic.

The online course was implemented in the *Moodle Stoa* extension area of the University of São Paulo (USP), available at http://cursosextensao.usp.br/course/view.php?id=133, and was evaluated by a group of specialists. After some suggested readjustments, the students accessed the course. In the virtual learning environment, the participants had access to eight tutors, experienced nurses in higher education in Nursing, Emergency and Distance Education (DE). During the online course, theoretical learning was virtually assessed by means of pre- and post-test scores. At the end of the theoretical study, the practical evaluation was scheduled on one of five days, from 8am to 6pm, in December of 2014 and also in February of 2015, a period related to data collection. The skills were evaluated in classes using simulated practice, in the Nursing Skills Laboratory (NSL) of the university.

After previous familiarization with the scenario and equipment, the students, in pairs, administered BLS with an *automated external defibrillator* (AED), in simulation of care for the adult with cardiopulmonary arrest, during four minutes in the extra-hospital environment. They were evaluated by the researcher and another tutor on the use of printed checklists and electronic feedback devices. At the end of the simulation, a *debriefing* was performed, and the students were taken to another space, organized in a similar way to the previous one, for the purpose of reviewing techniques and eventual clarification of doubts, with guidance from the third tutor. The tutorials were oriented toward the management of equipment, as well as the role during evaluation of the simulation and the reinforcement activity. Previous experience as instructors in BLS courses and training of emergency professionals facilitated the understanding of the activity dynamics and the evaluator role.

For statistical analysis, means and standard deviations (SD) were calculated for pre- and post-test scores, course evaluations, and performances in the practical evaluation. Absolute and relative frequency was used for sex, age and motivation to participate. A significance level of 5% was adopted. Variables with non-normal distribution were analyzed using Kendal’s correlation coefficient. The difference between the post-test and pre-test scores was considered as a parameter for theoretical learning. A paired t-test was applied to test the hypothesis of difference between the pre- and post-test means; and analysis of variance (ANOVA) was used to evaluate theoretical learning. Linear regression was calculated to verify the association between theoretical learning (dependent variable), year in nursing school for each student, and whether they attended emergency classes before the online course (independent variables). The Statistical Package for the Social Sciences (SPSS), version 22, was used.

## Results

Among the 62 students who completed the online course, the majority (87%) was female, with a mean age of 21.47 years (SD 2.39). Of these, 90.3% were in the first and second years of undergraduate nursing courses, while 9.7% were in the third and fourth years. Regarding previous knowledge, 50% had not participated in an emergency class prior to the online course; 53.3% did not know BLS; 61.2% were familiar with the Moodle^®^ platform; 69.1% had not previously participated in the distance education classes.

As for digital fluency, 100% had access to the Internet, and for 98.9%, access was via cellphone. Regarding the motivation for taking the course, 96.8% targeted the practical applicability of learning. Since the participation in the course did not have evaluation of the students, the difference between the post-test and pre-test was considered as a parameter for theoretical learning, with a variation from zero to ten. From the pre- and post-test scores, the means of the scores were calculated in each phase of the evaluation. The mean in the pre-test was 6.4 (SD1.6) and, in the post-test, 9.3 (SD 0.82, p <0.001, N = 62). In the paired t-test, significant differences in the means of the pre- and post-test scores were identified, with an increase in the mean and a decrease in the SD in the post-test. Differences in the means of the scores in the pre- and post-test were analyzed in relation to the year of study in the undergraduate program, which were grouped two by two, the first and second year in one group, and the third and fourth year in another, as shown in Table 1.

**Table 1:** Description of scores in pre- and post-test, according to the year in which the students were enrolled in the nursing program. São Paulo, SP, Brazil, 2014-2015

**Year of course**	**Phase**	**Mean**	**Standard deviation**	**Confidence interval**		**p value**	
						**Phase**	**Interaction**
**1** ^**st**^ **and 2** ^**nd**^	**Pretest**	**6.19**	**1.59**	**5.76**	**6.61**	**<0.001**	**0.475**
	**Posttest**	**9.20**	**1.60**	**8.98**	**9.41**		
**3** ^**rd**^ **and 4** ^**th**^	**Pretest**	**7.17**	**0.83**	**5.87**	**8.46**		
	**Posttest**	**9.67**	**0.61**	**9.00**	**10.33**		

The ANOVA analysis showed significant differences in the means of pre-test scores among the 56 students in the first two years of the nursing course, and the group of six students in the last two years. The analysis showed that, after the online course, the increase in mean scores was significant (p <0.001), regardless of the year in which the students were enrolled in the nursing program. At the end of the online course, no significant difference (p = 0.475) was identified in learning among first and second year students, compared to third and fourth year students.

To identify the variables associated with learning, a multiple linear regression model with progressive inclusion (forward stepwise) was adjusted. The association was shown to be significant, and inversely proportional to learning, in relation to the enrolled year in the nursing school course (coefficient 0.542, PE 0.215, p=0.015), and participation of the student in an emergency class before the online course (coefficient 0.903; PE 0.437; p=0.044, R^2^=0.253).

During the practical evaluation of the simulated activity, the electronic records from the immediate feedback devices were used. The mean percentage of performance was 43.7%, which corresponded to basic CPR (from 0 to 49%); mean length of compression cycles per second of 20.5; correct positioning of the hands to perform compression of 93.2%; mean number of compressions of 167.2; mean depth of compressions of 48.1 millimeters; mean chest release of 100%; mean ventilation of 8.2 ventilations every two minutes; mean ventilation volume of 742.7 milliliters; percentage of flow fraction of 40.3%. In addition to the electronic devices for immediate feedback during the practical evaluation, the checklist records used to follow the student’s actions were also used to analyze the results (Table 2). 

**Table 2:** Description of the actions performed in practical simulation, according to the checklist. São Paulo, SP, Brazil, 2014-2015

**Actions**	**N**	**%**
**Total**	**62**	**100**
**Tap the person on the shoulders**	**55**	**88**
**Call out loud**	**56**	**90**
**Expose the chest**	**61**	**98**
**Check for breathing**	**60**	**97**
**Activate 192 (or local emergency service)**	**47**	**76**
Ask someone to get AED*	**57**	**92**
**Check for carotid or femoral pulse**	**48**	**77**
**Place the heel of one hand on the breastbone**	**53**	**87**
**Give 30 chest compressions**	**58**	**95**
**Press down about 5 cm into the chest**	**54**	**89**
**Let the chest rise completely**	**55**	**90**
**Open the airway**	**59**	**97**
**Give two rescue breaths**	**60**	**97**
Turn on the AED*	**58**	**97**
Apply AED* electrode pads to chest	**62**	**100**
Attach cables to AED*	**60**	**97**
**Verify that no one is touching the person for rhythm analysis**	**56**	**90**
**Verify that no one is touching the person before shocking**	**54**	**87**
**Push the “shock” button**	**61**	**98**
**Perform compressions again after shock.**	**55**	**89**

*Automated External Defibrillator

In the practical activity, according to the success of the 20 actions provided on the checklist, considering the score equivalent to 0.5 per item, the mean score was 9.1 (SD 0.95). The plausibility of the hypothesis was confirmed in this study, and a contribution to learning about BLS with the online course was verified.

## Discussion

The results of investigations and resuscitation guidelines converge upon the use of videos and online courses as resources in life support education^15^. Thus, the development of the online BLS course aimed at stimulating the participant’s autonomy, favoring the self-managed learning process in a self-instructional way, combining the assumptions of Andragogy and Significant Learning Theory with educational technologies, due to the profile of students who seek distance-learning courses. Most are adults whose previous experiences can be widely used, since learning is understood as a process of gaining knowledge and/or experience^13^. 

Among the results found in the evaluation of learning, the significant difference between the post-test and pre-test scores evidenced the learning gain in the theoretical study on BLS at the end of the online course, regardless of the students’ year in the undergraduation course, or previous participation in emergency classes. This course contributed to learning, which was evidenced by the significant increase in the mean of the scores, analyzed between the beginning and the end of the course.

The correlation among the variables aimed to analyze the outcomes related to learning, and to understand if the improvement in the learning achievement could be explained by different events, other than the online course, as an educational intervention. The correlations with learning were associated with the undergraduate course year of the student, and the fact that the student had previously attended emergency classes. Although these two variables influenced the best results in the pre-test, performed by the students in third and fourth year of nursing school, at the end of the online course, the students enrolled in the first and second year of nursing school achieved similar results, even without previous knowledge obtained in emergency classes.

The finding of another investigation indicated that nursing students acquired significant theoretical achievement at the end of the online course, when assessing the learning on BLS obtained in the virtual environment^16^.

Enhancement in theoretical and practical learning was identified, with improvement in performance, regardless of the type of strategy used. Learning outcomes obtained in traditional courses, those guided by instructors, and self-learning videos were similar. Because of this, computer-based courses associated with the practice of resuscitation maneuvers can be a reasonable alternative to benefit greater standardization, in addition to a probable reduction in time and resources^17^.

Another relevant aspect is the frequent approximation to the subject, which contributes to retention of learning of life support maneuvers, as knowledge tends to degrade over time. At the university where this study was conducted, the BLS subject is introduced to the students in the Critical Care Nursing of Adults and Elderly course, given in the third year of the nursing program. Respecting curricular programming, it is necessary to consider the opportunities for the subject to be presented to the students earlier, providing new possibilities to increase attendance and (re)training, either in the field of practice, in clinical training, or in educational activities on BLS, assigning new meaning to learning and lived experiences of the students.

The importance of frequent training is consensus. Short and frequent training courses are highly recommended; the greater the exposure to the content, the greater the retention and safety in applying life support principles^18^. Although theoretical learning reaches satisfactory levels at the end of the course or training, learning retention is of particular concern in relation to life support performance skills over the time elapsed after the educational intervention. In general, adult learning retention after training in resuscitation maneuvers varies from 50 to 60%^19^.

Considering that the knowledge retention about life support maneuvers occurs more effectively when verified in professionals, especially those working in emergency situations, or in those who apply the knowledge more frequently, it is necessary to consider strategies that favor the students’ learning, because they are less frequently exposed to emergency situations.

Recommendations regarding the use of simulation with high fidelity simulators and feedback devices corroborate the need for higher quality learning processes and results in education, for the practical application of resuscitation maneuvers^6^. Improvement in resuscitation maneuvers were also identified when using feedback devices in training of laypersons, conferring higher quality when performing BLS, either in the initial phase of training or in periodic review^20^.

Evidence from a systematic review, in 2009, indicated positive aspects in the use of devices of immediate feedback in the CPR maneuvers, which was also applicable for situations in real-time care, supporting learning and retention of learned knowledge and skills, with recommendations to investigate the impact on patient survival^12^. Subsequently, authors indicated that the combination of the use of feedback devices in training and real-time care in cases of extra-hospital CPR contributed positively to patient survival^21^.

Considering that short courses and regularity in attendance have a positive influence on learning, as no significant correlation with longer training time was found^22^, the online course is a viable resource in professional training and continuing education.

The convenience sample used was a limitation of this study, which was influenced by a period of work strikes (from May to September of 2015) and university stoppage, generating accumulation in student demand, and complicating the ability to participate in the research, performed without a practice pre-course phase. 

## Conclusion

The results showed an increase in the mean score of the post-test, which indicated a more significant learning gain for students in the first years, or for those who did not attend emergency classes previously.

The use of technological resources and mobile devices of immediate feedback to verify the students’ performance in the practical evaluation was a valuable support, with the measurement of aspects such as hand positioning and thorax release, which are generally evaluated subjectively by observation. These parameters, evaluated with the device, indicate the quality of the compressions during resuscitation, giving greater objectivity and precision in the evaluation process, in addition to encouraging the student to raise awareness about his/her performance of the care. The BLS online course enabled access to knowledge, acting as a knowledge space and an environment for reflection on emergency actions, stimulating clinical reasoning and decision-making. 

In conducting similar studies, practical activity is suggest, both at the beginning and at the end of the online course.
